# Fertility Outcomes in Men with Nonobstructive Azoospermia Due to Hypogonadotropic Hypogonadism After Gonadotropin Therapy

**DOI:** 10.3390/jcm15031204

**Published:** 2026-02-03

**Authors:** Athanasios Zachariou, Athanasios Zikopoulos, Eleftheria Markou, Sotirios Koukos, Grigorios Daligaros, Sotirios Skouros, Fotios Dimitriadis, Michael Chrisofos, Nikolaos Sofikitis, Aris Kaltsas

**Affiliations:** 1 Laboratory of Spermatology, Department of Urology, Faculty of Medicine, School of Health Sciences, University of Ioannina, 45110 Ioannina, Greece; azachariou@uoi.gr (A.Z.); sotiriskoukos@gmail.com (S.K.); gregdali@gmail.com (G.D.); sotirisskouros@hotmail.com (S.S.); 2 Royal Cornwall Hospitals NHS Treliske Truro, Foundation Trust UK, Truro TR1 3LJ, UK; thanzik92@gmail.com; 3 Department of Microbiology, University Hospital of Ioannina, 45500 Ioannina, Greece; eleftheria.markou4@gmail.com; 4 Department of Urology, Faculty of Medicine, School of Health Sciences, Aristotle University of Thessaloniki, 54124 Thessaloniki, Greece; helabio@yahoo.gr; 5 Third Department of Urology, Attikon University Hospital, School of Medicine, National and Kapodistrian University of Athens, 12462 Athens, Greece; mchrysof@med.uoa.gr (M.C.); ares-kaltsas@hotmail.com (A.K.)

**Keywords:** non-obstructive azoospermia, hypogonadotropic hypogonadism, microTESE, ICSI, male infertility, spermatogenesis

## Abstract

**Background/Objectives:** Hypogonadotropic hypogonadism (HH) is an uncommon but treatable cause of non-obstructive azoospermia (NOA). Fertility can often be restored with gonadotropin therapy. This study evaluated spermatogenic and reproductive outcomes in men with HH-related NOA managed by stepwise gonadotropin therapy, microdissection testicular sperm extraction (microTESE) for persistent azoospermia, and assisted reproduction when indicated. **Methods:** A retrospective cohort study included 35 men treated between 2010 and 2022. Human chorionic gonadotropin (hCG), with or without follicle-stimulating hormone (FSH), was administered to induce spermatogenesis. Outcomes included sperm appearance in the ejaculate, microTESE sperm retrieval rate in persistent azoospermia, and pregnancy and live birth outcomes after natural conception or in vitro fertilization with intracytoplasmic sperm injection (IVF-ICSI) when required. **Results:** Mean gonadotropin therapy duration was 12.0 months (range 6–24). Sperm appeared in the ejaculate in 27/35 men (77%). The remaining 8/35 (23%) underwent microTESE, with sperm retrieved in 7/8 (88%). Seven couples proceeded to IVF-ICSI, undergoing 11 cycles that yielded 6 clinical pregnancies (55% per cycle) and 5 live birth deliveries, including 2 twin pregnancies. Among responders, 13 natural pregnancies occurred, resulting in 13 live birth deliveries, including 2 twin pregnancies. Overall, 18/35 men (51%) achieved biological fatherhood, corresponding to 18 live birth delivery events (4 twin and 14 singleton deliveries) and 22 newborns. **Conclusions:** In men with HH-related NOA, exogenous gonadotropin therapy is expected to induce spermatogenesis in most patients. MicroTESE provides high sperm retrieval rates for those without ejaculatory sperm. Through an integrated approach of hormonal induction, microsurgical sperm retrieval, and assisted reproduction, approximately half of patients may ultimately achieve biological fatherhood in longer-term follow-up, depending on baseline severity and partner factors.

## 1. Introduction

Male factor infertility contributes to approximately half of infertility in couples [1]. Globally, infertility affects an estimated 10–15% of couples, and azoospermia is present in about 1% of men in the general population and in 10–20% of men evaluated for infertility [2]. Azoospermia is broadly classified as non-obstructive or obstructive. Non-obstructive azoospermia (NOA) reflects impaired spermatogenesis, whereas obstructive azoospermia results from blockage within the male reproductive tract [3]. Because azoospermia comprises heterogeneous etiologies with distinct management pathways, accurate phenotyping is essential for counseling and treatment selection [3].

NOA is most frequently attributable to primary testicular failure, including genetic and histopathological entities such as Klinefelter syndrome, Y chromosome microdeletions, maturation arrest, and Sertoli cell-only patterns [4]. A smaller subset is pre-testicular and results from endocrine disorders that reduce gonadotropin stimulation of an otherwise structurally intact testis [5]. Hypogonadotropic hypogonadism (HH) is a central cause within this category and is characterized by low serum testosterone with low or inappropriately normal luteinizing hormone (LH) and follicle-stimulating hormone (FSH) levels [5]. Recognition of HH in men with azoospermia is clinically important because, unlike most causes of NOA, it is often amenable to targeted hormonal therapy that can reestablish spermatogenesis and expand fertility options [6].

HH may be congenital or acquired. Congenital isolated HH, also termed gonadotropin-releasing hormone deficiency, includes Kallmann syndrome with anosmia and normosmic idiopathic HH [7]. Many affected men present with absent or incomplete pubertal development, small testes, low serum testosterone, and infertility in early adulthood. Cryptorchidism is common in congenital forms and may further compromise testicular development and future spermatogenic potential [8]. Acquired HH arises later in life and is linked to hypothalamic or pituitary disease or functional suppression, including pituitary lesions, hyperprolactinemia, head trauma, pituitary irradiation, and exogenous androgen exposure [9].

The diagnostic evaluation of azoospermia must distinguish central hypogonadism from primary testicular failure and obstructive etiologies because treatment strategy and prognosis differ substantially [10]. Clinical assessment relies on detailed history and targeted physical examination, with attention to pubertal development, prior androgen exposure, symptoms or signs of androgen deficiency, and associated features that suggest a congenital syndrome (e.g., anosmia or hyposmia) [10]. Genital examination remains integral, including assessment of testicular size and consistency and evaluation of the epididymides and vasa deferentia to identify findings consistent with obstruction [3]. Contemporary guidelines also recommend karyotyping and Y chromosome microdeletion screening as part of the evaluation of NOA to guide counseling and reproductive planning [11].

For men desiring fertility, management of HH differs fundamentally from testosterone replacement. Exogenous testosterone improves symptoms of androgen deficiency but does not induce spermatogenesis and can suppress the hypothalamic–pituitary–gonadal axis, thereby worsening fertility potential [12]. The standard fertility-directed approach is gonadotropin replacement therapy using human chorionic gonadotropin (hCG) as an LH analogue to stimulate Leydig cell testosterone production, with FSH activity to support Sertoli cell function and spermatogenesis [13]. In clinical practice, treatment often begins with hCG to establish adequate intratesticular testosterone, with FSH added when spermatogenic induction is incomplete or when testicular development is limited, particularly in prepubertal-onset disease [14]. Response to gonadotropin-induced spermatogenesis is heterogeneous, and time to response may be prolonged; factors that have been associated with treatment kinetics in prior series include testicular volume, history of cryptorchidism, and markers of Sertoli cell activity [15].

Even with endocrine induction, azoospermia can persist despite optimized therapy in a subset of men [16]. In such circumstances, surgical sperm retrieval and assisted reproductive techniques may be required. Microdissection testicular sperm extraction (microTESE) is widely regarded as the preferred retrieval approach for men with NOA because magnification facilitates identification of seminiferous tubules more likely to contain spermatogenesis while limiting unnecessary tissue excision [17]. In the setting of HH, endocrine stimulation before retrieval is biologically plausible as a means to promote focal spermatogenesis that may not be detectable in the ejaculate, and clinical reports have described microTESE after gonadotropin therapy in this population [18].

Despite the clinical logic of a staged pathway integrating diagnosis, endocrine induction, selective surgical retrieval, and assisted reproduction when needed, the literature remains fragmented and typically focuses on endocrine induction or surgical/ART components in isolation, often within small cohorts given the rarity of HH among men with NOA [19]. There is a need for consolidated data reflecting real-world clinical sequencing to guide evaluation, counseling, and stepwise management across endocrine, surgical, and assisted reproduction domains.

## 2. Materials and Methods

### 2.1. Study Design, Setting, and Study Period

This was a retrospective cohort study of adult men with HH and NOA who were evaluated and treated for infertility at the Department of Urology, University of Ioannina (Greece), between January 2010 and December 2022. Clinical, laboratory, and treatment data were extracted from institutional medical records.

### 2.2. Eligibility Criteria

Eligible patients were at least 18 years old, had azoospermia confirmed on at least two semen analyses including assessment of the centrifuged pellet, had biochemical findings consistent with hypogonadotropic hypogonadism, and expressed a desire for biological paternity.

**Figure 1:**
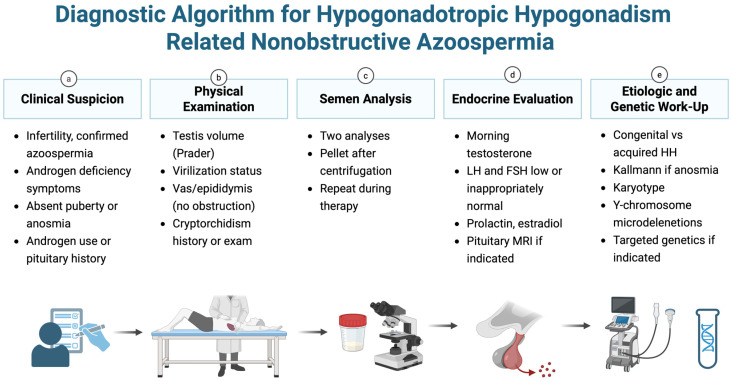
Diagnostic algorithm for hypogonadotropic hypogonadism–related non-obstructive azoospermia. The pathway summarizes the clinical, semen, endocrine, and etiologic/genetic evaluation used to confirm HH-related NOA, including pellet assessment, pituitary imaging when indicated, scrotal ultrasound, and karyotype plus Y-chromosome microdeletion testing. Created in BioRender. Kaltsas, A. (2026) https://BioRender.com/gxz8dxg (accessed on 28 January 2026). The letters (**a**–**e**) denote the sequential steps of the algorithm: (**a**) clinical suspicion; (**b**) physical examination; (**c**) semen analysis; (**d**) endocrine evaluation; and (**e**) etiologic and genetic work-up.

Patients were excluded if azoospermia was attributed to an obstructive cause such as vasectomy or congenital bilateral absence of the vas deferens; if baseline gonadotropins were elevated suggesting primary testicular failure; if there was prior gonadotoxic chemotherapy or radiotherapy; or if available documentation was insufficient to determine eligibility or ascertain key outcomes along the clinical pathway. The diagnostic work-up and classification approach used in this cohort are summarized in Figure 1.

### 2.3. Diagnostic Definitions

HH was defined as secondary hypogonadism characterized by low serum total testosterone (or testosterone in the low-normal range) with low or inappropriately normal LH and FSH levels, in contrast to the elevated gonadotropins typical of primary testicular failure. Institutional reference ranges provided by the Clinical Biochemistry Laboratory of the University Hospital of Ioannina at the time of testing were total testosterone 2.8–7.8 ng/mL (≈280–780 ng/dL), LH 1.2–8.6 IU/L, and FSH 1.3–19.3 IU/L.

HH was classified as congenital or acquired. Congenital HH was defined by absent or incomplete spontaneous puberty with persistent biochemical secondary hypogonadism and no alternative explanatory diagnosis. Kallmann syndrome was defined as congenital HH with anosmia or hyposmia. Acquired HH was defined by normal pubertal development followed by secondary hypogonadism due to a documented hypothalamic or pituitary disorder, or by idiopathic adult-onset secondary hypogonadism after exclusion of secondary causes.

Azoospermia was defined as complete absence of spermatozoa in the ejaculate on at least two separate semen analyses, each including examination of the centrifuged pellet. Semen analyses were performed in accordance with World Health Organization guidance. Pellet assessment was performed after high-speed centrifugation, approximately 3000× *g* for 15 min.

Spermatogenic response to gonadotropin therapy was operationally defined as the appearance of any spermatozoon in the ejaculate on at least one semen analysis during treatment. Extremely low concentrations (including cryptozoospermia with sperm detectable only in the centrifuged pellet) were classified as a positive response, consistent with definitions used in prior series [20]. Men with intermittent sperm presence on some analyses but not others were still classified as responders, acknowledging that very low sperm output may fluctuate and that documentation of any sperm reflects partial restoration of spermatogenesis. Persistent azoospermia on all follow-up semen evaluations despite gonadotropin therapy defined a non-response; these men were counseled regarding options including surgical sperm retrieval and assisted reproduction.

Clinical pregnancy was defined as ultrasound documentation of an intrauterine gestational sac with fetal cardiac activity. Live birth delivery was defined as delivery resulting in at least one live-born infant. Biological fatherhood was defined as the male patient having sired one or more live-born offspring. Newborn count referred to the number of live-born infants, with multiples counted individually.

### 2.4. Baseline Clinical and Laboratory Assessment

At presentation, patients underwent standardized clinical evaluation including medical and reproductive history with emphasis on pubertal development, prior fertility, prior use of exogenous androgens, comorbidities, and prior genitourinary surgery. Physical examination focused on genital findings. Testicular volume was assessed using a Prader orchidometer and recorded in milliliters. Testis position, consistency, and the presence of masses were documented. Secondary sexual characteristics and clinical signs of hypogonadism were recorded. The vas deferens and epididymides were assessed to support a non-obstructive phenotype.

Baseline endocrine testing included morning total testosterone, LH, FSH, prolactin, estradiol, and additional pituitary axes when clinically indicated. Pituitary magnetic resonance imaging of the sellar region was performed when suggested by clinical context (e.g., neurologic symptoms, visual complaints, or hyperprolactinemia).

Genetic evaluation was performed in all patients and included karyotype analysis and Y chromosome microdeletion testing in men with NOA, in accordance with guideline-based practice. Additional targeted genetic assessments were performed when clinically indicated by the phenotype.

Baseline variables included demographic characteristics, etiology classification of hypogonadotropic hypogonadism, history of cryptorchidism, prior androgen exposure, baseline hormonal profile, and baseline testicular volume. Treatment variables included gonadotropin regimen selection, dose escalation patterns as recorded, and duration of therapy.

### 2.5. Gonadotropin Treatment Protocol and Monitoring

Gonadotropin therapy was offered as first-line treatment to induce spermatogenesis in men with HH-associated azoospermia. Treatment was initiated with hCG to provide LH activity. A typical starting regimen was 1500–2000 IU administered subcutaneously two to three times weekly [21]. The hCG dose was adjusted based on serial morning total testosterone measurements (typically every 4–6 weeks), with the aim of achieving mid-normal serum total testosterone concentrations of approximately 450–600 ng/dL (4.5–6.0 ng/mL). This target falls within the adult male reference range (2.8–7.8 ng/mL) and was selected to avoid supraphysiologic testosterone levels [22]. Treatment-related adverse effects potentially related to gonadotropins were documented in the medical record.

Semen analyses (including centrifuged pellet assessment when sperm were not seen on direct microscopy) were performed at regular intervals, approximately every 3 months, to identify the first appearance of sperm in the ejaculate [23]. In men who remained azoospermic after an adequate trial of optimized hCG monotherapy for approximately 3–6 months, with testosterone normalized on therapy, FSH was added to provide combined gonadotropin stimulation [22]. The timing of FSH supplementation was guided by standardized clinical criteria, including persistent azoospermia despite normalized testosterone, baseline gonadotropin levels interpreted against the institutional adult male reference ranges (LH 1.2–8.6 IU/L and FSH 1.3–19.3 IU/L) to confirm central HH, and baseline indicators of disease severity such as absent or incomplete pubertal development and very small testicular volume, approximately 4 mL or less, in whom earlier combined therapy may be warranted. FSH was administered either as human menopausal gonadotropin or recombinant FSH, typically starting at 75 IU subcutaneously two to three times weekly and escalated up to 150 IU two to three times weekly based on predefined dose escalation according to semen analysis response and tolerability [21]. Combined therapy was continued until sperm appeared in the ejaculate or until a shared decision was made to proceed to surgical sperm retrieval in cases of persistent azoospermia despite prolonged treatment, which may extend to 12–24 months in selected patients. Any prior use of exogenous testosterone was documented and discontinued prior to initiation of gonadotropin therapy.

### 2.6. Surgical Sperm Retrieval

Men with persistent azoospermia despite an adequate course of gonadotropin therapy (generally at least 6–12 months of maximal hormone doses achieving mid-normal testosterone levels) were offered surgical sperm retrieval. The indication for microTESE was NOA remaining after prolonged gonadotropin stimulation at the highest tolerated doses. All microTESE procedures were performed by the same microsurgical team under general anesthesia, following the standard microdissection technique [24]. The testis with larger volume was typically explored first to determine the operative sequence; bilateral microTESE was routinely performed in all patients, regardless of sperm retrieval from the initial side. The tunica albuginea was opened widely (e.g., via equatorial incision) to expose the parenchyma, and under an operating microscope (approximately 16–25× magnification) the seminiferous tubules were systematically inspected. Dilated, opaque-appearing tubules (which are more likely to contain active spermatogenesis) were selectively identified and gently extracted using fine forceps, thereby minimizing tissue excision while maximizing sperm yield [24]. The excised tubule samples were immediately handed off to an embryologist for examination. MicroTESE was systematically performed bilaterally in all cases. In this study, sperm retrieval success was defined as the identification of at least one viable spermatozoon suitable for intracytoplasmic sperm injection (ICSI) during the microTESE procedure—i.e., any recovered testicular sperm for potential use was considered a successful retrieval [22]. Hemostasis was meticulously maintained throughout (using bipolar cautery on small vessels as needed) and the tunica albuginea was closed with fine non-absorbable sutures once the microdissection was completed on each side. No conventional (blind) testicular sperm extraction was performed in this cohort; microTESE was the chosen method for all surgical sperm retrievals, reflecting contemporary best practice for NOA due to its higher sperm yield and lower tissue loss compared to traditional techniques [24].

### 2.7. Histopathological Evaluation

In all microTESE cases, a portion of testicular tissue was sent for histopathological examination as per institutional protocol. To minimize removal of potentially productive seminiferous tubules, the diagnostic specimen was obtained at the end of the procedure as a small, non-targeted (random) fragment of testicular parenchyma, preferentially from tissue not required for sperm search (e.g., parenchyma at the incision margin) and, when available, from residual tissue remaining after laboratory processing. No additional systematic ‘mapping’ biopsies were performed solely for histology. The specimen was evaluated by the institutional pathology department, including a genitourinary pathologist, to screen for germ cell neoplasia in situ and to characterize the spermatogenic pattern. Histological findings were categorized using standard criteria into diagnoses such as Sertoli cell–only syndrome (complete absence of germ cells), maturation arrest (presence of germ cells that halt at an early or mid-stage of spermatogenesis), or hypospermatogenesis (all stages present but in reduced quantity), based on the predominant cell types and stages observed in the biopsy [25]. This routine diagnostic biopsy approach also served to exclude intratubular germ cell neoplasia or other testicular pathology, which is especially pertinent given the elevated baseline risk of in situ malignancy in some infertility patients (e.g., those with a history of cryptorchidism or severe testicular atrophy) [25]. The histopathological results provided an adjunct classification of spermatogenic impairment for each patient, but therapeutic decisions in this study were primarily guided by the clinical sperm retrieval outcomes described above.

### 2.8. Assisted Reproductive Technology

Couples in whom sperm became available—either in the ejaculate during gonadotropin therapy or via microTESE—were counseled regarding their reproductive options. The decision to attempt natural conception versus to proceed with ART was individualized based on the quantity/quality of the available sperm (for example, extremely low sperm counts or poor motility), relevant female partner factors, and the couple’s own preferences. Female partner age and any documented female-factor infertility diagnoses were extracted when available from clinical records and collaborating ART center reports, but were not systematically available for all couples.

When testicular sperm were utilized, fertilization was performed by in vitro fertilization with intracytoplasmic sperm injection (IVF-ICSI), in accordance with standard practice for severe male-factor infertility and the use of surgically retrieved sperm [22]. IVF-ICSI was also recommended in cases where ejaculated sperm were present but in very low numbers or demonstrated poor motility such that conventional insemination or unassisted conception was considered unlikely.

For each ART cycle, key outcomes—including whether a clinical pregnancy was achieved and whether it culminated in a live birth—were documented based on records from the collaborating ART center and follow-up in our hospital. If surplus sperm and/or sperm-bearing testicular tissue was obtained (either from ejaculate or microTESE), cryopreservation was performed using standard protocols with cryoprotectant to facilitate subsequent ICSI attempts without repeat surgical retrieval and to optimize treatment scheduling [26]. Cryopreserved samples were stored in liquid nitrogen and thawed for use in later IVF-ICSI cycles as needed.

### 2.9. Study Objectives and Endpoints

This study evaluated the proportion of men with HH-related NOA who achieved biological fatherhood when managed within a stepwise pathway of gonadotropin therapy, microTESE for persistent azoospermia, and assisted reproduction when indicated. The primary endpoint was biological fatherhood, defined as the patient siring ≥1 live-born offspring; delivery-level outcomes included the number of live birth delivery events (delivery resulting in ≥1 live-born infant) and the total newborn count (multiples counted individually). Key secondary endpoints were: (i) spermatogenic response to gonadotropin therapy, defined as detection of any spermatozoon in the ejaculate on ≥1 semen analysis during treatment (including sperm detectable only in the centrifuged pellet); (ii) microTESE sperm retrieval success among men with persistent azoospermia, defined as identification of ≥1 viable spermatozoon suitable for intracytoplasmic sperm injection; and (iii) IVF-ICSI outcomes among cycles using surgically retrieved sperm, including clinical pregnancy (ultrasound documentation of an intrauterine gestational sac with fetal cardiac activity) and live birth delivery. Follow-up was defined as the interval from initiation of gonadotropin therapy to the last documented clinical contact; for men achieving biological fatherhood, time-to-event was calculated to the first live birth delivery event.

### 2.10. Statistical Analysis

Continuous variables are summarized as mean ± standard deviation (SD) (with ranges reported where informative) or as median [interquartile range (IQR)] for variables with non-normal distributions or where median-based reporting was clinically more interpretable. Categorical variables are summarized as counts and percentages. Analyses were primarily descriptive, and inferential testing was undertaken in an exploratory, hypothesis-generating framework. For exploratory between-group comparisons (responders vs. nonresponders), continuous variables were compared using the Mann–Whitney U test. Categorical variables were compared using two-sided exact tests: Fisher’s exact test for 2 × 2 tables and the Fisher–Freeman–Halton exact test for larger contingency tables. All tests were two-sided, and *p*-values are reported. A *p*-value < 0.05 was considered to indicate potential statistical significance; no adjustments were made for multiple comparisons because analyses were hypothesis-generating. Given the modest sample size and small subgroup sizes, between-group comparisons have limited statistical power and precision, and *p*-values may be unstable. Accordingly, interpretation emphasizes the magnitude and uncertainty of observed differences rather than statistical significance alone. Given the modest sample size and limited number of outcome events, multivariable modeling was not performed to avoid overfitting and unstable estimates. Missing data were not imputed, and denominators are indicated where applicable. Statistical analyses were performed using IBM SPSS Statistics (IBM Corp., Armonk, NY, USA; version 25).

### 2.11. Ethics

The study protocol was approved by the Institutional Review Board as a retrospective review of de-identified patient data and was conducted in accordance with the Declaration of Helsinki and relevant institutional policies. Written informed consent for clinical treatment and for use of clinical data for research was obtained according to institutional practice.

## 3. Results

### 3.1. Patient Characteristics

A total of 35 men with azoospermia due to hypogonadotropic hypogonadism treated between 2010 and 2022 were included. The mean age at treatment initiation was 38.9 ± 5.1 years (range 30–48). Baseline hormone levels reflected marked hypogonadotropic hypogonadism (total testosterone 87.7 ± 50.3 ng/dL) with low gonadotropins. Mean testicular volume was 5.8 ± 3.9 mL. A history of cryptorchidism was present in 12 men (34%). All 35 patients underwent comprehensive genetic testing (karyotype analysis and Y-chromosome microdeletion screening); all had a 46, XY karyotype with no AZF microdeletions. During therapy, 27 men (77%) were classified as responders and 8 (23%) as nonresponders based on the detection of sperm in the ejaculate. Baseline characteristics stratified by responder status are summarized in Table 1.

Testicular volume differed between responders and nonresponders (*p* = 0.03), with a greater mean testicular volume in responders than in nonresponders (7.7 ± 4.6 vs. 4.5 ± 2.9 mL, Table 1). Given the small nonresponder subgroup (*n* = 8) and multiple unadjusted comparisons, this finding should be interpreted cautiously as hypothesis-generating and should not be used to infer an independent effect or to define a clinical decision threshold. However, this pattern is biologically plausible, as larger baseline testicular volume is generally associated with greater spermatogenic potential in men with HH undergoing gonadotropin therapy. A history of cryptorchidism was more frequent among nonresponders (4/8, 50%) than responders (8/27, 30%), although this difference was not statistically significant (Table 1). This trend may partially confound the observed testicular volume difference, because prior cryptorchidism can impair testicular development and future spermatogenic potential [8]. For the remaining baseline variables, the between-group comparisons did not provide strong evidence of differences in these exploratory analyses (Table 1); however, clinically meaningful differences cannot be excluded because of limited statistical power and imprecise estimates.

### 3.2. Response to Gonadotropin Therapy

The mean duration of gonadotropin therapy in the full cohort was 12.0 ± 5.2 months (range 6–24 months). Sperm were detected in the ejaculate during therapy in 27/35 men (77%). The remaining 8/35 men (23%) remained azoospermic despite therapy and proceeded to microTESE.

Among responders, the median time to first sperm detection in the ejaculate was 9 [6–15] months (Table 2). Peak sperm concentrations remained low overall (median 5 [2–12] million/mL), and very low sperm output was common, including cryptozoospermia in 3/27 men (11%) (Table 2). According to the predefined pathway, hCG was initiated in all patients, and FSH was added when ejaculatory sperm were not detected after an initial treatment interval. Among responders, 12/27 (44%) achieved sperm detection during the hCG only phase, typically within 6 to 7 months, supporting an initial trial of hCG monotherapy in selected men with milder HH phenotypes (e.g., partial pubertal development and/or relatively larger baseline testes) who demonstrate early testosterone rise and testicular growth on treatment. The remaining 15/27 responders (56%) had FSH added and subsequently demonstrated sperm in the ejaculate during combined therapy. Men with more severe, prepubertal-onset phenotypes—very small testes (≈≤4 mL), complete lack of pubertal development, and/or a history of cryptorchidism—appear less likely to achieve spermatogenesis with hCG alone and may warrant earlier addition of FSH to provide direct Sertoli-cell stimulation [20]. Pragmatically, clinicians may start hCG alone in appropriate patients and escalate to combined hCG + FSH if no sperm are detected despite sustained mid-normal testosterone and minimal testicular growth after ~6 months, while individualizing timing according to fertility goals and couple time constraints. By the end of treatment, all nonresponders had received combined hCG plus FSH without induction of ejaculatory sperm. Treatment-related adverse effects were documented in 3/35 men (9%) during gonadotropin therapy. These events were mild, managed conservatively, and did not lead to treatment discontinuation, and included transient gynecomastia (*n* = 1), localized injection-site reactions (*n* = 1), and mood irritability (*n* = 1). No serious or long-term complications were recorded.

Natural conception occurred during or shortly after therapy in 13 responder couples, resulting in 13 live birth deliveries, including two twin gestations. The remaining 14 responder couples had not achieved pregnancy by the end of follow-up and were still attempting spontaneous conception at last documented contact. In several cases, follow-up after first sperm detection was short. Female partner data were not consistently recorded; when available, partner age was typically in the early 30s (range mid-20s to early 40s), and female-factor issues such as advanced maternal age or endometriosis were occasionally documented. These factors may have influenced pregnancy achievement despite ejaculatory sperm detection in some responder couples.

The median follow-up from initiation of gonadotropin therapy to last documented contact was 30 months (interquartile range 18 to 48, overall range 6 to 96 months). Median follow-up was 24 months in responders and 36 months in nonresponders. Among men who achieved biological fatherhood, the median time from treatment initiation to first live birth delivery event was 32 months. Table 2 summarizes key endocrine and semen parameters observed during gonadotropin therapy.

### 3.3. Sperm Retrieval by microTESE

Eight men who remained azoospermic after gonadotropin therapy underwent microTESE. Sperm were retrieved in 7/8 cases (88%). Among the seven retrieval-positive cases, testicular histopathology showed hypospermatogenesis in five and late maturation arrest in two. The single retrieval-negative patient had congenital GnRH deficiency and a history of cryptorchidism. Despite prolonged combined hCG plus FSH stimulation, bilateral microTESE did not identify spermatozoa, and histopathology demonstrated a Sertoli cell-only pattern. No perioperative complications were noted in the surgical records. Table 3 summarizes microTESE surgical and histological outcomes for nonresponders.

### 3.4. Assisted Reproduction Outcomes

Seven couples (20% of the cohort) proceeded to IVF-ICSI. All of these couples were those in which the male partner required microTESE, and no couples with sperm detected in the ejaculate initiated IVF with ICSI during the study period, which reflected a couple-driven decision after counseling rather than a center-specific policy. In responders, including those with cryptozoospermia, ART was discussed as an option, but couples generally opted to continue attempts at natural conception once any ejaculated sperm became available, with ART deferred at last follow up. Female partner factors and sperm cryopreservation decisions were not systematically captured in the retrospective records, and therefore cannot be reliably disentangled as drivers of this pattern; however, all documented ART cycles in this cohort were performed using microTESE derived sperm. In all IVF-ICSI attempts, surgically retrieved testicular sperm were used. Sperm obtained via microTESE were cryopreserved in most cases, enabling a total of 11 IVF-ICSI cycles across the seven couples (median 2 [range 1–2] cycles per couple). Across the 11 IVF-ICSI cycles, 6 clinical pregnancies were achieved (55% per cycle). At last available follow-up, five of these six clinical pregnancies resulted in live birth delivery events (including two twin pregnancies), whereas one clinical pregnancy did not culminate in a live birth delivery event within the study follow-up and was therefore not counted as a live birth delivery in the dataset.

Combining natural and assisted conception outcomes, 18/35 men (51%) achieved biological fatherhood. These 18 men accounted for 18 live birth delivery events, including 4 twin and 14 singleton deliveries, resulting in 22 newborns. Table 4 provides an integrated summary of reproductive outcomes for the full cohort.

**Figure 2:**
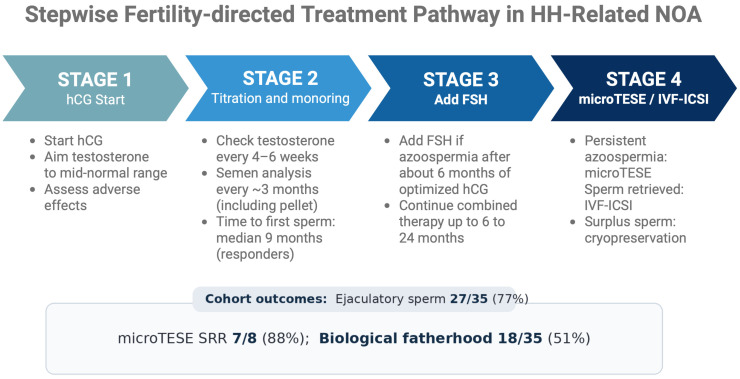
Stepwise fertility-directed treatment pathway in hypogonadotropic hypogonadism–related non-obstructive azoospermia. The schematic summarizes hCG initiation with dose titration, FSH addition in persistent azoospermia, escalation to microTESE, and use of retrieved sperm for IVF–ICSI when required, with cohort-level outcomes shown. Created in BioRender. Kaltsas, A. (2026) https://BioRender.com/617cszb (accessed on 28 January 2026).

Taken together, these findings illustrate a coherent stepwise pathway from hormonal induction to surgical sperm retrieval and assisted reproduction when required. Most men achieved ejaculatory sperm with gonadotropin therapy, and microTESE provided an effective second-line option for persistent azoospermia. At the cohort level, this integrated strategy resulted in biological fatherhood for approximately half of the treated men within the available follow-up. The stepwise pathway and key outcomes are summarized in Figure 2.

## 4. Discussion

In this cohort of men with HH-related NOA, a stepwise approach integrating hormonal therapy and microsurgical sperm retrieval yielded notable long-term fertility outcomes. Exogenous gonadotropin treatment induced spermatogenesis (appearance of sperm in semen) in 77% of patients, and microTESE provided sperm in 88% of those who remained azoospermic despite hormones. Ultimately, 51% of the men became biological fathers, resulting in 18 live-birth delivery events (22 offspring). These findings highlight that azoospermia due to HH, unlike primary testicular failure, is often reversible with appropriate endocrine stimulation, whereas microsurgical sperm retrieval offers an effective adjunct when ejaculated sperm cannot be achieved [27]. Gonadotropin deficiency represents a treatable form of male infertility, in contrast to intrinsic testicular failure where therapeutic options are limited to sperm retrieval or donor sperm [27].

The spermatogenic success rate observed here is consistent with prior reports in the literature. Larger series and reviews have documented that approximately 60–80% of men with congenital or acquired hypogonadotropic hypogonadism will develop sperm in the ejaculate after gonadotropin therapy [27,28]. Differences in reported response rates likely reflect varying cohort characteristics, such as severity of gonadotropin deficiency, presence of cryptorchidism, prior pubertal development, and the intensity and duration of treatment [28,29]. In this cohort, the mean duration of therapy was 12 months (range 6–24), underlining the prolonged time course often required for induction of spermatogenesis. This is in line with previous analyses showing a median of roughly 12–18 months to first sperm appearance, and that men with more severe presentations (e.g., history of cryptorchidism or prepubertal HH) may require up to 24 months or longer of treatment to achieve sperm output [28,30].

The overall profile of the present cohort—profoundly low endogenous gonadotropins, very low testosterone levels, and small testicular volumes—is characteristic of severe HH, a phenotype that has been associated with slower or less robust responses to gonadotropin therapy in prior studies [28,29]. Consistent with the literature, larger baseline testicular volume and the absence of cryptorchidism have been reported as favorable factors for achieving spermatogenesis, whereas very small testes (e.g., <4 mL) and a history of undescended testes are more frequently associated with delayed and/or less reliable sperm appearance in the ejaculate [28,29]. In the present cohort, responders had larger baseline testicular volumes than nonresponders (7.7 ± 4.6 vs. 4.5 ± 2.9 mL), and cryptorchidism was numerically more frequent among nonresponders (50% vs. 30%), mirroring these previously described associations. Collectively, these observations support the concept that baseline testicular development reflects the severity and timing of gonadotropin deficiency, which may influence both the probability and the time course of spermatogenic recovery under therapy [16,30]. From a counseling perspective, men with marked testicular underdevelopment or other markers of long-standing HH should be advised that longer treatment durations may be required and that early response is less certain [16,30]. Although these subgroup comparisons are exploratory and limited by small numbers, they support pragmatic counseling that very small testicular volume and a history of cryptorchidism may be associated with a more prolonged course and a lower likelihood of achieving ejaculatory sperm, even if focal spermatogenesis may still be amenable to microTESE.

Accordingly, treatment duration and dosing are best individualized based on baseline clinical characteristics and early treatment response. Men with prepubertal-onset (congenital) HH—characterized by absent puberty, very small testes, and often a history of cryptorchidism—generally require longer therapy with earlier intensification, whereas patients with adult-onset or less severe HH (larger baseline testes) may achieve spermatogenesis more quickly on hCG alone. Serial morning serum testosterone measurements guide hCG dose titration to physiologic levels, and semen analyses (including pellet microscopy) are performed at regular intervals to monitor for sperm appearance; some clinicians also track Sertoli cell markers (e.g., inhibin B or AMH) as adjuncts to gauge testicular activity, although these were not utilized in this cohort [31]. If early testicular growth or hormonal response is inadequate in a severe case, the regimen can be escalated (for example, by adding FSH or increasing the hCG dose) rather than prolonging subtherapeutic dosing, while counseling that spermatogenesis induction may take many months in such patients. Conversely, if no sperm are detected after an extended trial (e.g., beyond 18–24 months), diminishing returns should be considered and a shared decision made whether to continue hormonal therapy or proceed to microTESE and assisted reproduction.

For the subset of men who remained azoospermic despite maximal hormone stimulation, the findings in this cohort underscore the value of microdissection sperm retrieval. Sperm were successfully retrieved by microTESE in 7 of 8 such men, corresponding to a high retrieval rate of 88%. This aligns with reports that even when gonadotropin therapy fails to produce ejaculated sperm, many HH patients still have focal areas of spermatogenesis within the testes that can be harvested surgically [32]. Notably, testicular histopathology in all seven successful cases showed active spermatogenesis (most commonly hypospermatogenesis, and in two cases late maturation arrest), whereas the single failed retrieval corresponded to a Sertoli-cell-only pattern on biopsy. This distribution of histologic patterns is biologically plausible: exogenous gonadotropins often induce heterogeneous or patchy germ cell development in previously azoospermic testes, such that some seminiferous tubules produce sperm even if overall counts remain undetectable in semen [33]. Indeed, contemporary guidelines emphasize that a diagnostic biopsy is of limited value in NOA because any given histological pattern (even Sertoli-cell-only) might coexist with focal sperm production elsewhere in the testis [33]. This concept is also supported by contemporary clinical data showing that diagnostic testicular biopsy has limited predictive value for sperm retrieval outcomes in NOA [34].

The present results support this notion, demonstrating that microTESE can locate spermatozoa in men who would otherwise be considered “complete” failures of medical therapy. At the same time, the number of nonresponders in this series was small, and all patients had received extensive hormonal priming before surgery. Caution is therefore warranted in generalizing the exact probability of microTESE success in untreated HH patients or in drawing firm conclusions about which histologic subtypes have the best prognosis. Nonetheless, the present data add to the evidence that microTESE is a highly effective next step for HH patients after an adequate trial of gonadotropin therapy [32,33]. When spermatogenesis is biochemically induced but not reflected in the ejaculate, microdissection allows targeted retrieval from any pockets of sperm production, offering these men a path to paternity that would not exist without surgical intervention [32,33].

The reproductive outcomes following sperm acquisition in this cohort are encouraging but should be interpreted with appropriate context. Among seven couples who proceeded to IVF-ICSI using microTESE-derived sperm, a 55% clinical pregnancy rate per cycle (6 pregnancies in 11 cycles) and five live births were observed. Additionally, thirteen unassisted (natural) pregnancies were achieved by couples where the male partner had sperm in the ejaculate, leading to healthy live births. These figures suggest that once sperm is available—either spontaneously or via retrieval—the chances for successful pregnancy are reasonably good. However, it is important to recognize that pregnancy and live birth rates are influenced by multiple factors beyond the male’s treatment. Female partner characteristics (such as age and ovarian reserve), the utilization and quality of assisted reproductive techniques, and the duration of follow-up all substantially affect these endpoints. In a large longitudinal analysis of men with hypogonadotropin deficiency, the presence of adverse female fertility factors was found to be the strongest determinant of pregnancy outcomes, more than halving the likelihood of conception and significantly prolonging time to pregnancy [27]. The observation of twin pregnancies in both the natural conception and IVF groups further illustrates how outcomes can be confounded by chance and clinical practice: spontaneous twin gestations occur stochastically, while in IVF settings the incidence of twins has been historically influenced by the number of embryos transferred [35]. These considerations underscore that the ultimate success of the treatment pathway (achieving a live birth) cannot be solely attributed to the restoration of spermatogenesis in the male. That restoration is often a necessary first step, but the probability of a couple taking home a baby will also depend on female reproductive health and the quality of embryo implantation and gestation [27]. For clinicians and patients, a balanced perspective is needed—gonadotropin therapy and microTESE can enable previously impossible opportunities for biological fatherhood, yet the final outcomes hinge on a host of other variables that must be managed and optimized in parallel. Finally, it should be acknowledged that a small subset of men may remain azoospermic despite both endocrine induction and microTESE (1/35 in this cohort). In this situation, management pathways are individualized through shared decision-making and may include continued or extended gonadotropin therapy when clinically appropriate and, in selected cases, consideration of repeat microTESE after counseling that accounts for testicular reserve and prior histology. Alternative family-building options such as donor sperm, embryo donation where legal and available, adoption, or choosing a child-free life can be discussed in a non-directive manner, and access to psychosocial support may be valuable.

Several limitations of this study should be acknowledged. The retrospective, single-center design may introduce selection bias, and treatment protocols were not fully uniform across the 12-year span of data, reflecting evolving practice patterns. The sample size (35 men) is relatively modest, limiting the statistical power to detect subtle differences or to identify independent predictors of success with confidence. Given the rarity of HH-related NOA, these findings should be considered hypothesis-generating and warrant confirmation in future multicenter, large-sample studies. In particular, collaborative prospective registries or harmonized multicenter cohorts with standardized gonadotropin protocols, uniform definitions of response, consistent documentation of female partner and ART variables, and longer follow-up would provide adequately powered estimates and more robust, generalizable inference regarding predictors and couple-dependent reproductive outcomes. Furthermore, not all patients had comprehensive baseline measurements recorded (for example, some lacked detailed hormonal profiling or testicular measurements before therapy), which constrains the ability to perform robust multivariable analysis on factors influencing outcomes. Future larger prospective cohorts and/or multicenter registries should evaluate candidate predictors spanning endocrine and clinical domains, including baseline and on-treatment testosterone, inhibin B, anti-Müllerian hormone (AMH), and gonadotropins where informative, alongside testicular volume, cryptorchidism history, pubertal development, and time to testicular growth on therapy. Genetic profiling could include variants associated with congenital HH (e.g., ANOS1, FGFR1, GNRHR, PROKR2) and, where appropriate, broader infertility-related gene panels [36]. These candidate predictors could be integrated into prespecified multivariable models to derive risk scores/nomograms with internal validation and external validation across centers, and may be complemented by standardized imaging metrics or microTESE histopathology patterns when available. The cohort was also etiologically heterogeneous: cases included congenital GnRH deficiency (with and without anosmia), post-pituitary surgery hypogonadism, and idiopathic forms of HH. Such heterogeneity, while reflective of real-world clinical practice, complicates the interpretation of pooled results, since each subgroup may have distinct prognoses and optimal management nuances. Additionally, data on female partner factors and assisted reproduction details were not collected in a standardized way for all couples. Maternal age, ovarian stimulation outcomes, and embryo quality were not systematically recorded, although these factors are central determinants of IVF success. Where documented, female partner age was in the early 30s, with a range spanning the mid-20s to the early 40s. In addition, clinical records occasionally noted gynecological comorbidities, including prior ovarian surgery and irregular menses suggestive of ovulatory dysfunction. Accordingly, the reported pregnancy and live birth outcomes should be interpreted as couple-dependent endpoints, reflecting the combined contribution of male spermatogenic restoration and sperm acquisition, female reproductive potential and comorbidities, and ART-related factors such as ovarian stimulation, embryo quality, and transfer practices. These outcomes should therefore not be interpreted as isolated measures of male treatment efficacy, and pregnancy or live birth rates from this cohort should be contextualized by partner and procedural factors when available to avoid misclassification in future systematic reviews and meta-analyses. Future studies should prospectively capture partner age, infertility diagnosis, duration of infertility, ovarian reserve markers such as AMH and/or antral follicle count (AFC), and prior ART history. When IVF is performed, cycle-level variables should be recorded, including stimulation response and embryo transfer parameters (number and stage of embryos transferred, fresh versus frozen transfer), and embryo testing status when applicable. Analyses should stratify outcomes by female age bands (e.g., <35, 35–37, 38–40, ≥41 years) and adjust for female age and female-factor infertility. Outcomes should be reported per couple and, when relevant, per ART cycle to avoid misinterpretation of couple-dependent endpoints. Finally, follow-up was variable and, in some cases, relatively short in the context of fertility (median 30 months [IQR 18–48], range 6–96 months), and some men may have achieved pregnancies after the study period. These limitations highlight the need for cautious interpretation and suggest that future prospective studies or multicenter collaborations could provide stronger evidence to guide patient counseling.

Within the above constraints, the present study provides an integrated view of the therapeutic strategy for HH-related infertility, encompassing hormonal induction, microsurgical sperm retrieval, and assisted reproduction. The outcomes support the clinical paradigm of using gonadotropin therapy as first-line treatment for men with HH and NOA, with microTESE offered as a secondary intervention for those who do not achieve sperm in the ejaculate [33,37]. This stepwise approach is well-aligned with contemporary guideline recommendations, which emphasize that men with HH should be treated with gonadotropins (hCG with the addition of FSH as needed) to stimulate spermatogenesis, rather than being directly routed to donor sperm or empirical surgeries [37]. In contrast to other causes of NOA where medical therapy has limited utility, HH represents a unique scenario where hormonal therapy can restore natural fertility in a substantial proportion of patients [36]. The data in this cohort further show that if hormonal therapy alone is insufficient, proceeding to microTESE can yield a high chance of sperm retrieval, enabling the use of IVF-ICSI to achieve pregnancy. Thus, about half of all treated men in this series realized the goal of biological fatherhood, an outcome that underscores the importance of recognizing HH promptly and managing it aggressively with the available modalities. As always, patient counseling should temper optimism with realism: success rates are influenced by individual circumstances, and achieving a live birth often requires persistence through a combination of endocrine, surgical, and reproductive techniques. Nevertheless, the overall message is a positive one—through an individualized and multidisciplinary treatment algorithm, even men with initially complete azoospermia from HH have a reasonable likelihood of ultimately fathering children [27,38].

## 5. Conclusions

In men with NOA attributable to HH, this retrospective single-center cohort describes a stepwise pathway based on gonadotropin-induced spermatogenesis followed by microTESE in persistent azoospermia, in which biological fatherhood was achieved in a substantial proportion of couples. Most men developed ejaculatory sperm during hormonal therapy, and sperm retrieval remained high among those requiring microTESE. When sperm was available for natural conception or IVF with intracytoplasmic sperm injection, clinically meaningful pregnancy and live birth outcomes were achieved across the cohort. These findings may be consistent with the use of an integrated endocrine, microsurgical, and assisted reproduction strategy in appropriately selected men with HH-related NOA. Further studies with larger cohorts and standardized protocols are warranted to refine treatment duration, define optimal sequencing of interventions, and clarify patient and partner factors associated with successful reproductive outcomes.

## Figures and Tables

**Table 1:** Baseline characteristics and treatment parameters in men with complete baseline data (*n* = 35), stratified by responder status. Responders are men with sperm detected in the ejaculate during therapy.

Characteristic	Total (*n* = 35)	Responders (*n* = 27)	Nonresponders (*n* = 8)	*p*-Value ᵃ
Age, years	38.9 ± 5.1 (30–48)	38.5 ± 5.0 (30–45)	40.3 ± 5.5 (34–48)	0.30
Baseline LH, IU/L	0.2 [0.05–0.9]	0.3 [0.1–1.1]	0.1 [0.01–0.4]	0.58
Baseline FSH, IU/L	0.6 [0.2–1.8]	0.7 [0.3–2.0]	0.4 [0.1–1.2]	0.33
Baseline total testosterone, ng/dL	87.7 ± 50.3	96.9 ± 40.6	81.5 ± 56.8	0.49
Total testosterone after treatment, ng/dL	517.8 ± 224.6	602.6 ± 173.9	466.0 ± 243.5	0.17
Mean testicular volume, mL	5.8 ± 3.9	7.7 ± 4.6	4.5 ± 2.9	0.03
Etiology of hypogonadotropic hypogonadism, *n* (%)				0.98
– Congenital GnRH deficiency with anosmia	8 (23%)	6 (22%)	2 (25%)	
– Pituitary tumor or surgery-related	4 (11%)	3 (11%)	1 (12%)	
– Idiopathic isolated hypogonadotropic hypogonadism	23 (66%)	18 (67%)	5 (62%)	
History of cryptorchidism, *n* (%)	12 (34%)	8 (30%)	4 (50%)	0.40
Duration of gonadotropin therapy, months	12.0 ± 5.2	11.7 ± 5.5	14.1 ± 5.0	0.27
Any treatment-related adverse effect, *n* (%)	3 (9%)	2 (7%)	1 (13%)	0.55

ᵃ *p*-values from Mann–Whitney U tests (continuous variables) and exact tests for categorical variables (Fisher’s exact test for 2 × 2 tables; Fisher–Freeman–Halton exact test for larger contingency tables), comparing responders vs. nonresponders. Continuous variables are presented as mean ± SD (range shown for age) or median [IQR], as indicated. Abbreviations: FSH, follicle-stimulating hormone; GnRH, gonadotropin-releasing hormone; LH, luteinizing hormone.

**Table 2:** Endocrine and semen response outcomes during gonadotropin therapy.

	Total (*n* = 35)	Responders (*n* = 27)	Nonresponders (*n* = 8)
Total testosterone after treatment, ng/dL	517.8 ± 224.6	602.6 ± 173.9	466.0 ± 243.5
Achieved testosterone ≥300 ng/dL, *n* (%)	31 (89%)	26 (96%)	5 (63%)
Time to first sperm in ejaculate, months	–	9 (6–15)	–
Peak sperm concentration, million/mL	–	5 (2–12)	–
Cryptozoospermia (sperm only after centrifugation), *n* (%)	–	3 (11%)	–
Severe oligozoospermia (<1 million/mL), *n* (%)	–	5 (19%)	–
Oligozoospermia (1–15 million/mL), *n* (%)	–	19 (70%)	–

Values are presented as mean ± standard deviation or median (IQR), as appropriate. Responders are defined as men with sperm detected in the ejaculate during therapy.

**Table 3:** MicroTESE and histopathology outcomes in nonresponders.

Outcome	Result
Nonresponders undergoing microTESE	8
Sperm retrieved	7 of 8
Sperm retrieval rate	7/8 (88%)
Histopathology pattern, *n* (%)	
Hypospermatogenesis	5 (63%)
Late maturation arrest	2 (25%)
Sertoli cell only (microTESE negative case)	1 (12%)
Perioperative complications, *n* (%)	0 (0%)
ART linkage	
Couples proceeding to IVF-ICSI after microTESE, *n* (% of microTESE cases)	7 (88%)
Number of IVF-ICSI cycles using surgically retrieved sperm, *n* (median [range] per couple)	11 (median 2 (1–2))

Nonresponders are men who remained azoospermic during gonadotropin therapy. microTESE: microdissection testicular sperm extraction. Histopathology patterns are reported per microTESE case (note that the Sertoli-cell-only pattern corresponds to the single sperm retrieval–negative case). ART: assisted reproductive technology. Abbreviations: IVF—in vitro fertilization; ICSI—intracytoplasmic sperm injection.

**Table 4:** Integrated reproductive outcomes in the full cohort of men treated (*n* = 35).

Outcome	Result
Mean duration of gonadotropin therapy	12.0 ± 5.2 months (range 6–24)
Sperm in ejaculate during gonadotropin therapy	27 of 35 men (77%)
Men proceeding to microdissection testicular sperm extraction	8 of 35 men (23%)
Sperm retrieved by microdissection testicular sperm extraction	7 of 8 cases (88%)
Couples proceeding to IVF-ICSI	7 couples (20% of cohort)
Number of IVF-ICSI cycles	11
Clinical pregnancies after IVF-ICSI	6 (55% per cycle)
Live birth deliveries after IVF-ICSI	5 (including 2 twin pregnancies)
Natural pregnancies among responders	13
Live birth deliveries from natural pregnancies	13 (including 2 twin pregnancies)
Biological fatherhood achieved	18 of 35 men (51%)
Total live birth delivery events	18
Delivery composition	4 twin and 14 singleton deliveries
Total newborns	22

Abbreviations: IVF, in vitro fertilization. ICSI, intracytoplasmic sperm injection.

## Data Availability

Data supporting the reported results are available from the corresponding author upon reasonable request.

## References

[1] Leslie S.W., Soon-Sutton T.L., Khan M.A.B. (2025). Male Infertility. StatPearls.

[2] Gudeloglu A., Parekattil S.J. (2013). Update in the evaluation of the azoospermic male. Clinics.

[3] Wosnitzer M., Goldstein M., Hardy M.P. (2014). Review of Azoospermia. Spermatogenesis.

[4] Tharakan T., Luo R., Jayasena C.N., Minhas S. (2021). Non-obstructive azoospermia: Current and future perspectives. Fac. Rev..

[5] Dimitriadis F., Adonakis G., Kaponis A., Mamoulakis C., Takenaka A., Sofikitis N., Simoni M., Huhtaniemi I.T. (2017). Pre-Testicular, Testicular, and Post-Testicular Causes of Male Infertility. Endocrinology of the Testis and Male Reproduction.

[6] Kumar R. (2013). Medical management of non-obstructive azoospermia. Clinics.

[7] Dode C., Hardelin J.P. (2009). Kallmann syndrome. Eur. J. Hum. Genet..

[8] Rodprasert W., Virtanen H.E., Makela J.A., Toppari J. (2019). Hypogonadism and Cryptorchidism. Front. Endocrinol..

[9] Fraietta R., Zylberstejn D.S., Esteves S.C. (2013). Hypogonadotropic hypogonadism revisited. Clinics.

[10] Oates R. (2012). Evaluation of the azoospermic male. Asian J. Androl..

[11] Brannigan R.E., Hermanson L., Kaczmarek J., Kim S.K., Kirkby E., Tanrikut C. (2024). Updates to Male Infertility: AUA/ASRM Guideline (2024). J. Urol..

[12] Crosnoe L.E., Grober E., Ohl D., Kim E.D. (2013). Exogenous testosterone: A preventable cause of male infertility. Transl. Androl. Urol..

[13] Boeri L., Capogrosso P., Salonia A. (2021). Gonadotropin Treatment for the Male Hypogonadotropic Hypogonadism. Curr. Pharm. Des..

[14] Zacharin M., Sabin M.A., Nair V.V., Dabadghao P. (2012). Addition of recombinant follicle-stimulating hormone to human chorionic gonadotropin treatment in adolescents and young adults with hypogonadotropic hypogonadism promotes normal testicular growth and may promote early spermatogenesis. Fertil. Steril..

[15] Cangiano B., Goggi G., Federici S., Bresesti C., Cotellessa L., Guizzardi F., Vezzoli V., Duminuco P., Persani L., Bonomi M. (2021). Predictors of reproductive and non-reproductive outcomes of gonadotropin mediated pubertal induction in male patients with congenital hypogonadotropic hypogonadism (CHH). J. Endocrinol. Investig..

[16] Liu P.Y., Baker H.W., Jayadev V., Zacharin M., Conway A.J., Handelsman D.J. (2009). Induction of spermatogenesis and fertility during gonadotropin treatment of gonadotropin-deficient infertile men: Predictors of fertility outcome. J. Clin. Endocrinol. Metab..

[17] Kaltsas A., Stavros S., Kratiras Z., Zikopoulos A., Machairiotis N., Potiris A., Dimitriadis F., Sofikitis N., Chrisofos M., Zachariou A. (2024). Predictors of Successful Testicular Sperm Extraction: A New Era for Men with Non-Obstructive Azoospermia. Biomedicines.

[18] Chen Y.K., Huang I.S., Chen W.J., Huang C.Y., Ho C.H., Huang E.Y., Huang W.J. (2021). Reproductive outcomes of microdissection testicular sperm extraction in hypogonadotropic hypogonadal azoospermic men after gonadotropin therapy. J. Assist. Reprod. Genet..

[19] Ide V., Vanderschueren D., Antonio L. (2020). Treatment of Men with Central Hypogonadism: Alternatives for Testosterone Replacement Therapy. Int. J. Mol. Sci..

[20] Dwyer A.A., Raivio T., Pitteloud N. (2015). Gonadotrophin replacement for induction of fertility in hypogonadal men. Best Pract. Res. Clin. Endocrinol. Metab..

[21] Rastrelli G., Vignozzi L., Maggi M. (2016). Different Medications for Hypogonadotropic Hypogonadism. Endocr. Dev..

[22] Schlegel P.N., Sigman M., Collura B., De Jonge C.J., Eisenberg M.L., Lamb D.J., Mulhall J.P., Niederberger C., Sandlow J.I., Sokol R.Z. (2021). Diagnosis and treatment of infertility in men: AUA/ASRM guideline part II. Fertil. Steril..

[23] Ortac M., Hidir M., Salabas E., Boyuk A., Bese C., Pazir Y., Kadioglu A. (2019). Evaluation of gonadotropin-replacement therapy in male patients with hypogonadotropic hypogonadism. Asian J. Androl..

[24] Dabaja A.A., Schlegel P.N. (2013). Microdissection testicular sperm extraction: An update. Asian J. Androl..

[25] Dohle G.R., Elzanaty S., van Casteren N.J. (2012). Testicular biopsy: Clinical practice and interpretation. Asian J. Androl..

[26] Amer M., Fakhry E. (2021). Fresh vs frozen testicular sperm for assisted reproductive technology in patients with non-obstructive azoospermia: A systematic review. Arab. J. Urol..

[27] Gialouris J.V., Conway A.J., Idan A., Savkovic S., Hermosilla R., Muir C.A., Bacha F., Zhang T., Hou W., Jayadev V. (2025). Efficacy of Gonadotropin Therapy to Induce Spermatogenesis and Fertility in Men with Pathologic Gonadotropin Deficiency. J. Clin. Endocrinol. Metab..

[28] Liu Z., Mao J., Wu X., Xu H., Wang X., Huang B., Zheng J., Nie M., Zhang H. (2016). Efficacy and Outcome Predictors of Gonadotropin Treatment for Male Congenital Hypogonadotropic Hypogonadism: A Retrospective Study of 223 Patients. Medicine.

[29] Miyagawa Y., Tsujimura A., Matsumiya K., Takao T., Tohda A., Koga M., Takeyama M., Fujioka H., Takada S., Koide T. (2005). Outcome of gonadotropin therapy for male hypogonadotropic hypogonadism at university affiliated male infertility centers: A 30-year retrospective study. J. Urol..

[30] Dwyer A.A., McDonald I.R., Quinton R. (2024). Current landscape of fertility induction in males with congenital hypogonadotropic hypogonadism. Ann. N.Y. Acad. Sci..

[31] Sinisi A.A., Esposito D., Maione L., Quinto M.C., Visconti D., De Bellis A., Bellastella A., Conzo G., Bellastella G. (2008). Seminal anti-Mullerian hormone level is a marker of spermatogenic response during long-term gonadotropin therapy in male hypogonadotropic hypogonadism. Hum. Reprod..

[32] Esteves S.C., Achermann A.P.P., Miyaoka R., Verza S., Fregonesi A., Riccetto C.L.Z. (2024). Clinical factors impacting microdissection testicular sperm extraction success in hypogonadal men with nonobstructive azoospermia. Fertil. Steril..

[33] Katz D.J., O'Donnell L., McLachlan R.I., Moss T.J., Boothroyd C.V., Jayadev V., Catford S.R. (2025). The first Australian evidence-based guidelines on male infertility. Med. J. Aust..

[34] Kaltsas A., Markou E., Zachariou A., Dimitriadis F., Symeonidis E.N., Zikopoulos A., Mamoulakis C., Tien D.M.B., Takenaka A., Sofikitis N. (2023). Evaluating the Predictive Value of Diagnostic Testicular Biopsy for Sperm Retrieval Outcomes in Men with Non-Obstructive Azoospermia. J. Pers. Med..

[35] Practice Committee of the American Society for Reproductive Medicine, the Practice Committee for the Society for Assisted Reproductive Technology (2021). Guidance on the limits to the number of embryos to transfer: A committee opinion. Fertil. Steril..

[36] Boehm U., Bouloux P.M., Dattani M.T., de Roux N., Dode C., Dunkel L., Dwyer A.A., Giacobini P., Hardelin J.P., Juul A. (2015). Expert consensus document: European Consensus Statement on congenital hypogonadotropic hypogonadism--pathogenesis, diagnosis and treatment. Nat. Rev. Endocrinol..

[37] Minhas S., Boeri L., Capogrosso P., Cocci A., Corona G., Dinkelman-Smit M., Falcone M., Jensen C.F., Gul M., Kalkanli A. (2025). European Association of Urology Guidelines on Male Sexual and Reproductive Health: 2025 Update on Male Infertility. Eur. Urol..

[38] Jungwirth A., Giwercman A., Tournaye H., Diemer T., Kopa Z., Dohle G., Krausz C., European Association of Urology Working Group on Male Infertility (2012). European Association of Urology guidelines on Male Infertility: The 2012 update. Eur. Urol..

